# PGC-1α and ERRα in patients with endometrial cancer: a translational study for predicting myometrial invasion

**DOI:** 10.18632/aging.103611

**Published:** 2020-09-13

**Authors:** LiLi Chen, XiaoDan Mao, MeiMei Huang, HuiFang Lei, LiFang Xue, PengMing Sun

**Affiliations:** 1 Laboratory of Gynecological Oncology, Fujian Maternity and Child Health Hospital, Affiliated Hospital of Fujian Medical University, Fuzhou 350001, Fujian, P.R. of China; 2 Reproductive Center, Fujian Maternity and Child Health Hospital, Affiliated Hospital of Fujian Medical University, Fuzhou 350001, Fujian, P.R. of China; 3 Department of Gynecology, Fujian Maternity and Child Health Hospital, Affiliated Hospital of Fujian Medical University, Fuzhou 350001, Fujian, P.R. of China; 4 Fujian Key Laboratory of Women and Children's Critical Diseases Research, Fujian Maternity and Child Health Hospital, Affiliated Hospital of Fujian Medical University, Fuzhou 350001, Fujian, P.R. of China

**Keywords:** PGC-1α, ERRα, epithelial-mesenchymal-transition, invasion, endometrial cancer

## Abstract

Background: PGC-1α and ERRα are closely related to tumor formation and progression. However, the mechanism underlying the involvement of PGC-1α/ERRα in regulating invasion and migration in endometrial cancer remains to be explored.

Results: Elevated levels of PGC-1α and ERRα were associated with advanced myometrial invasion, and PGC-1α and Vimentin expression was related to the depth of myometrial invasion in premenopausal endometrial cancer. Silencing of PGC-1α reduced ERRα activation and inhibited epithelial-mesenchymal-transition phenotypes, resulting in significant inhibition of invasion and migration. Overexpression of ERRα led to enhanced PGC-1α expression and increased activity of TFEB, promoting epithelial-mesenchymal-transition in endometrial cancer cells.

Conclusions: PGC-1α and ERRα induce the epithelial-mesenchymal-transition therefore invasion and migration in endometrial cancer, and may be novel biomarkers to predict the risk of advanced myometrial invasion.

Methods: PGC-1α, ERRα, and vimentin expression was analyzed in tissue microarrays using immunohistochemistry. PGC-1α and ERRα expression in endometrial cancer cell lines was investigated using quantitative PCR and western blotting analyses after infection with lentivirus-mediated small interfering RNA (siRNA) targeting PGC-1α (siRNA-PGC-1α) or overexpressing ERRα. E-cadherin and vimentin levels were determined using western blotting and cell immunouorescence analyses. Cell migration and invasiveness were evaluated using scratch and trans-well chamber assays.

## INTRODUCTION

Endometrial cancer is one of the most common malignancies in women. The American Cancer Society has estimated there would be 61,880 new cases and 12,160 deaths from endometrial cancer in 2019 [1]. The 5-year survival rate of endometrial cancer is 80-90% owing to early diagnosis and treatment, although 78.9% of the patients at surgically staged endometrial cancer have high risk of nodal metastases, and therefore poor prognosis [2]. Tumor invasion and metastasis are important biological characteristics of malignant tumors and to prevent/reduce them is key for the success of the clinical treatment. Studies show that the epithelial-to-mesenchymal transition (EMT) and its reverse mesenchymal-to-epithelial transition (MET) play crucial roles in the metastatic dissemination of carcinomas [3]. EMT is a cellular process loosely defined as a loss of the epithelial traits of tight cell–cell adhesion and apico-basal polarization and a gain of mesenchymal traits of motility and invasion [4]. EMT is characterized by the loss of E-cadherin, ZO-1, cytokeratin, as well as the upregulation of matrix metalloproteinase, fibronectin, α-smooth muscle actin, vimentin, snail, and slug [5]. These proteins have been defined as EMT biomarkers and, among them, E-cadherin, and vimentin are the most important biomarkers for epithelial and mesenchymal cells, respectively.

The occurrence and development of endometrial cancer is closely related to the imbalance of estrogen. In recent years, it has been found that estrogen-related receptor α (ERRα) is involved in the complex signal transduction of estrogen owing to the similarity between its structure and that of estrogen receptor (ER), and therefore plays a role in hormone-related tumors [6]. ERRα is regulated by the activity of peroxisome proliferator-activated receptor γ coactivator-1α (PGC-1α) [7], which is involved in cell proliferation, differentiation and development, and carcinogenesis [8]. Several studies have suggested that the PGC-1α/ERRα signaling pathway has important clinical significance in the expression of prostate cancer [9], colon cancer [10], breast cancer [11, 12], and other malignant tumors. The PGC-1α/ERRα signal is associated with tumor cell energy metabolism [13], angiogenesis, and invasion [14]. However, the mechanism of the PGC-1α/ERRα pathway in endometrial cancer invasion and metastasis remains to be explored. Therefore, this study investigated the levels of PGC-1α and ERRα in endometrial cancer tissues and cells. Deregulation of PGC-1α or ERRα expression were utilized to investigate the functional relationship between PGC-1α and ERRα. Our data aim to demonstrate that whether PGC-1α and ERRα are involved in the regulation of EMT via TFEB, and affect invasion and migration of endometrial cancer.

## RESULTS

### PGC-1α and ERRα positively correlate with more advanced myometrial invasion in endometrial cancer

**Figure 1:**
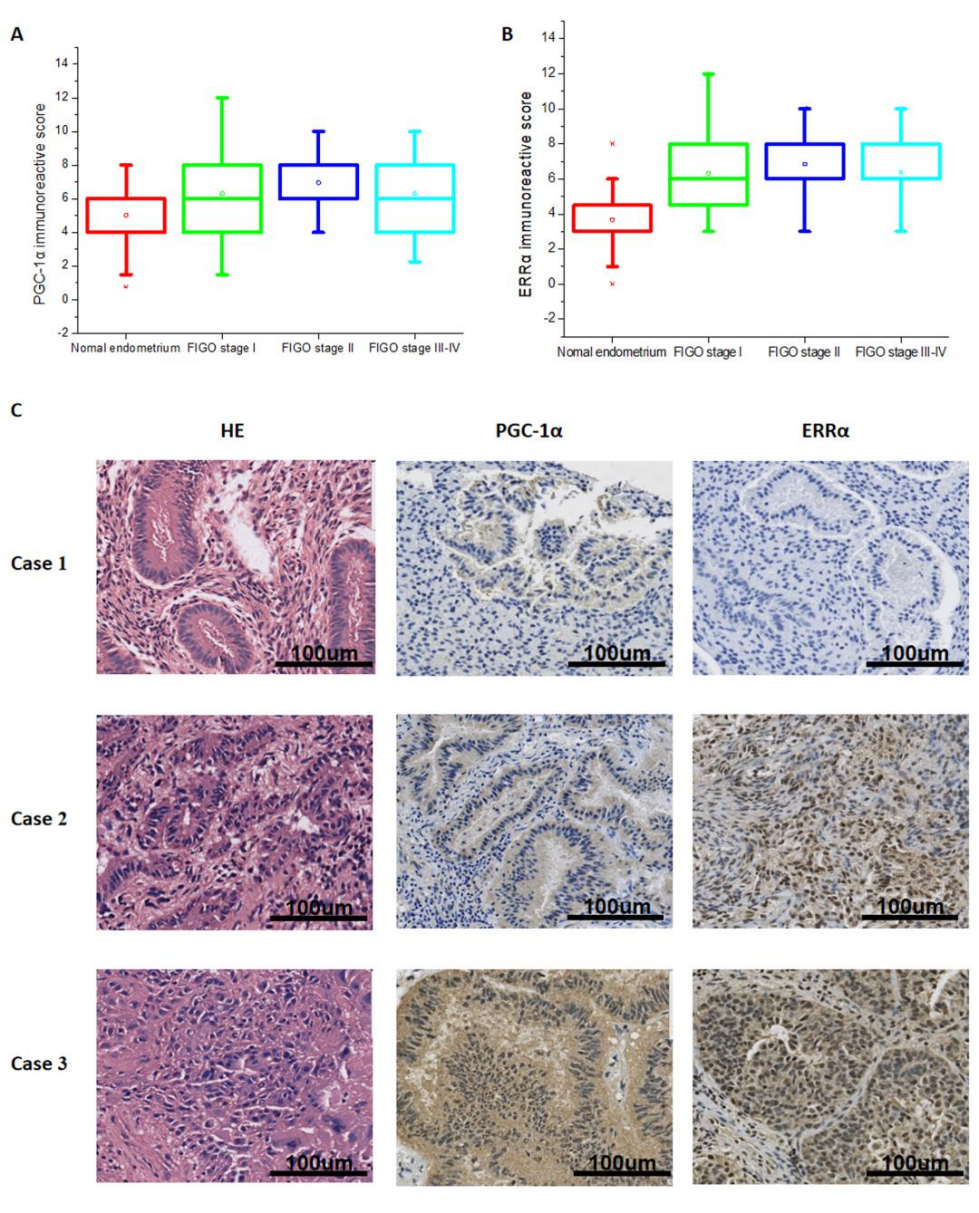
**Expression of PGC-1α and ERRα in normal endometrium and endometrial cancer tissue.** Expression of PGC-1α (**A**) and ERRα (**B**) in normal endometrium and different clinical stage endometrial cancer. (**C**) HE staining and immunohistochemical staining of PGC-1α, ERRα in normal endometrium (Case 1), superficial myometrial invasion EC (Case 2) and deep myometrial invasion EC (Case 3).

A total of 121 specimens from patients with endometrial cancer (N=81) or healthy subjects (N=40) were analyzed, and we found that PGC-1α was expressed in all tissue specimens examined. ERRα showed positive expression in all endometrial carcinoma samples and in 38 of 40 samples of normal endometrium. The immunoreactive score revealed that PGC-1α and ERRα expression was significantly higher in endometrial carcinoma than in normal endometrium (Figure 1A, 1B). Spearman’s correlation analysis showed that the expression of PGC-1α significantly associated with that of ERRα (Spearman’ s rank correlation 0.638, P < 0.001). We also analyzed the correlation between the expression of PGC-1α and ERRα and the clinicopathologic features of the subjects including FIGO stage, histologic grade, histology type, myometrial invasion, and nodal metastasis (Table 1). Higher expression of PGC-1α and ERRα positively correlated with more advanced myometrial invasion (P = 0.038 and 0.039, respectively). It was confirmed that the expression of PGC-1α and ERRα was higher in highly invasive endometrial cancer tissues than in less invasive endometrial cancer and significantly higher than in normal tissues (Figure 1C).

**Table 1:** Correlation between expression of PGC-1α /ERRα and clinicopathologic features.

		**PGC-1α**				**ERRα**		
	**+**	**++**	**+++**	**P**	**+**	**++**	**+++**	**P**
All case	41	66	14		45	64	10	
Normal endometrium	18	22	0		29	9	0	
Cancers	23	44	14		16	55	10	
FIGO stage								
I	17	25	9	0.793	13	31	7	0.407
II	1	9	3		1	11	1	
III-IV	5	10	2		2	13	2	
Grade								
I	7	19	6	0.181	5	22	5	0.121
II	11	15	5		9	18	4	
III	2	3	0		2	3	0	
Histology								
Endometrioid adenocarcinoma	18	36	11	0.928	15	41	9	0.127
Non- adenocarcinoma	5	8	3		1	14	1	
Myometrial invasion								
<1/2	19	30	7	0.038*	15	35	6	0.039*
≥1/2	4	14	7		1	20	4	
Nodal metastasis								
Negative	19	39	13	0.549	14	49	8	0.985
Positive	4	4	1		2	6	1	

Data between different groups were compared using χ^2^ tests. *mean *P*<0.05

### Analysis of factors related to advanced myometrial invasion in endometrial cancer

EC patients were divided into two groups: less and more than 1/2 myometrial invasion. Clinical characteristics were compared between the two groups, and no significant differences were observed in age, BMI, triglyceride, cholesterol, or electrolyte levels. In contrast, the serum glucose (P = 0.0407) and CA125 (P = 0.034) levels were higher in patients who had advanced myometrial invasion (Table 2). Factors such as blood glucose, serum CA125, PGC-1α, and ERRα with a P value of less than 0.05 after a t test or χ2 test were used as variables for single-factor logistic regression analysis. The results show that PGC-1α and ERRα were cogent predictors for myometrial invasion in patients with endometrial cancer (P <0.05) (Table 3).

**Table 2:** Characteristics of the less than 1/2 myometrial invasion group and more than 1/2 myometrial invasion group.

	**<1/2 myometrial invasion**	**≥1/2 myometrial invasion**	**P**
Age	52.57±8.119	55.80±5.568	0.0750
BMI	24.2652 ±3.3186	22.9268±2.9496	0.1171
Triglyceride	1.6120 ±1.5212	1.4824±0.9257	0.6951
Blood glucose	6.1525±2.6525	6.7071±2.0816	0.0407*
Total cholesterol	4.7443±1.0538	4.7046±0.9906	0.8763
Total protein TP	67.5464±9.2055	69.0200±11.0415	0.5337
Albumin ALB	40.0232±4.2653	40.4320 ±4.5290	0.6969
Globulin GLB	27.1875±6.0447	28.5880±8.4964	0.4001
Apolipoprotein a	1.1961±0.2169	1.2356±0.2463	0.4698
Apolipoprotein b	0.9332±0.3595	0.9052±0.2801	0.7308
Sodium ion	141.1309±2.1100	140.880±3.2187	0.6790
Potassium ion	4.0143±0.3255	4.0160±0.4210	0.9841
Calcium ion	2.2138±0.1789	2.2089±0.1812	0.9112
CA125	37.8600±63.1702	100.0522±270.0347	0.0340*
CA153	12.7551±7.8968	20.6720±42.0164	0.2035
CA199	61.2841±177.3394	468.0732±1568.5861	0.2385
SCC	1.2260±1.1609	1.7609±1.1061	0.0677
AFP	2.6731±1.2115	3.0200±1.3111	0.2609
CEA	1.8996±1.0804	2.3596±1.3041	0.1110

Numerical data are presented as the mean ± SD. Differences between two means were compared using Student’s t-test. *mean *P*<0.05.

**Table 3:** Logistic regression analysis of myometrial invasion in endometrial cancer.

**Factors**	**β**	**SE**	**P**	**OR**	**95%CI**
Blood glucose	0.084	0.094	0.371	1.088	0.904-1.309
CA125	0.003	0.003	0.290	1.003	0.997-1.009
PGC-1α	1.558	0.767	0.042	4.750	1.056-21.360
ERRα	0.947	0.471	0.044	2.579	1.025-6.490

Logistic regression analysis was used to identify the possible risk factors for myometrial invasion.

SE, standard error; OR, odds ratio. CI, confidence interval.

### Myometrial invasion in pre- and postmenopausal endometrial cancer is related to PGC-1α and vimentin

In this study, the frequency of deep 1/2 myometrial invasion of premenopausal endometrial cancer was 10/41 (24.4%), which was lower than that of postmenopausal endometrial cancer (15/40; 37.5%). Vimentin is an important indicator for cancer invasion and metastasis. Vimentin and PGC-1α expression levels were statistically different in pre- and postmenopausal endometrial cancer (P = 0.006 and P= 0.030, respectively). Spearman correlation analysis showed that the expression of PGC-1α in endometrial cancer was positively correlated with that of vimentin (r= 0.263, P = 0.018). Based on menopausal status combined with the depth of myometrial invasion of endometrial cancer, it was found that the expression of PGC-1α was related to the depth of myometrial invasion of premenopausal endometrial cancer (P = 0.022), but not to that of postmenopausal endometrial cancer (P = 0.056) (Table 4). Similarly, vimentin expression was related to the depth of myometrial invasion of premenopausal endometrial cancer (P = 0.009), but not to that of postmenopausal endometrial cancer (P = 0.064). Spearman correlation analysis showed that the expression of PGC-1α in premenopausal endometrial cancer patients was positively correlated with that of vimentin (r= 0.344, P = 0.027) (Table 4).

**Table 4:** PGC-1α and vimentin in different myometrial invasion of pre- and post-menopausal endometrial cancer.

	**PGC-1α**					**ERRα**					**Vimentin**			
	**+**	**++**	**+++**	**P**		**+**	**++**	**+++**	**P**		**-**	**+**	**++**	**+++**	**P**
Premenopausal EC															
<1/2 myometrial invasion	7	19	5	0.022		7	20	4			4	15	9	3	0.009
≥1/2 myometrial invasion	2	2	6		1	5	4	0.085		0	2	4	4
Postmenopausal EC															
<1/2 myometrial invasion	12	11	2	0.056		8	15	2			12	8	4	1	0.064
≥1/2 myometrial invasion	2	12	1		0	15	0	0.128		4	3	7	1

Data between different groups were compared using χ^2^ tests. EC, endometrial cancer.

### PGC- 1α and ERRα are mutually regulated in endometrial cancer cells

**Figure 2:**
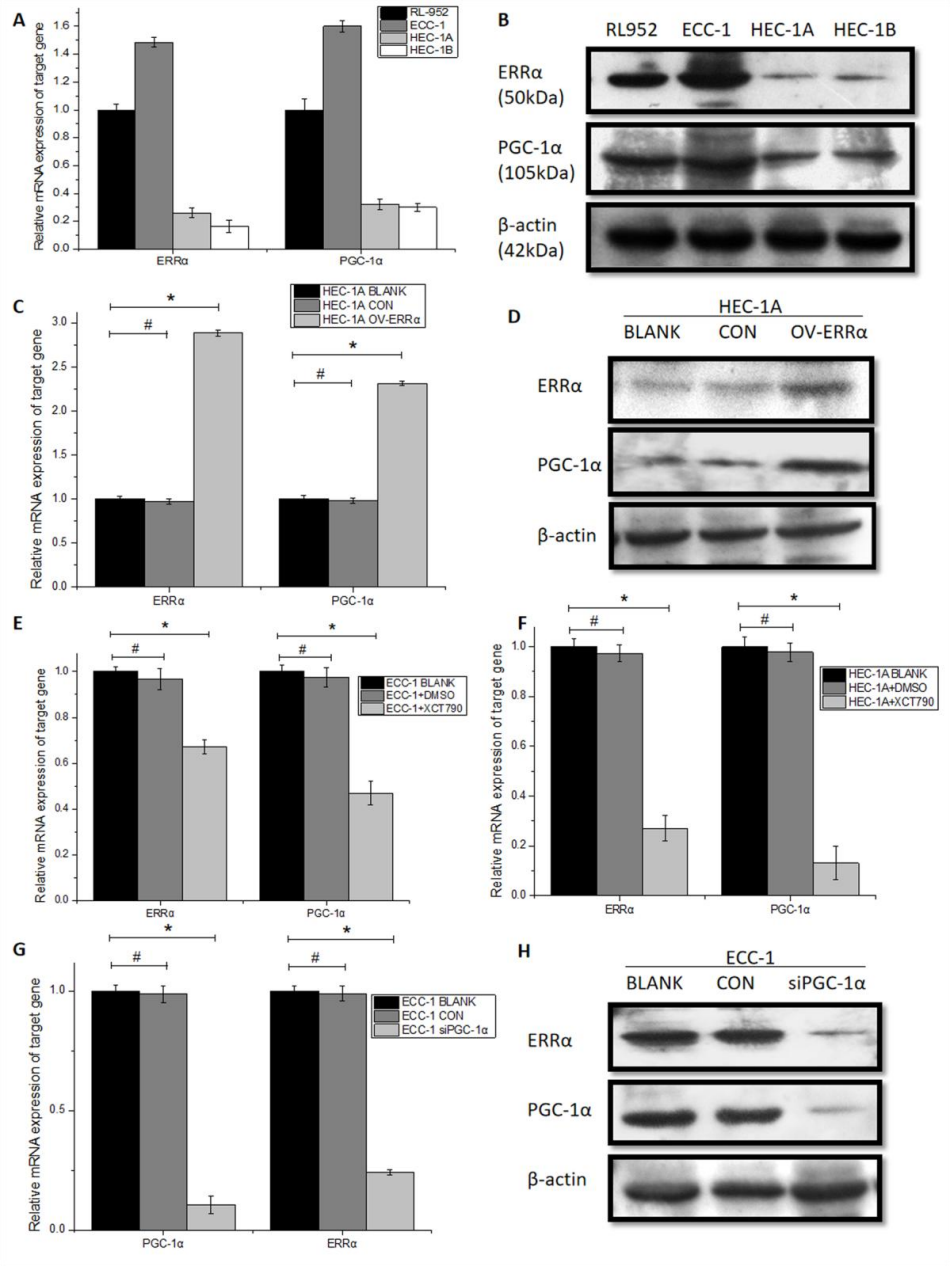
**Expression of PGC-1α and ERRα in endometrial cancer cells.** (**A**) related mRNA expression and (**B**) protein expression pattern of PGC-1α and ERRα in RL-952, ECC-1, HEC-1A and HEC-1B endometrial cancer cells. (**C**) PGC-1α and ERRα mRNA expression and (**D**) protein levels was significantly up-regulated after the infection with lentivirus targeted on OV-ERRα. Relative mRNA expression in ECC-1 (**E**) and HEC-1A (**F**) cells treated with XCT790 showed a downregulation of ERRα. (**G**) PGC-1α and ERRα mRNA expression (**H**) protein levels was significantly down-regulated after the infection with PGC-1α-siRNA, ERRα is consistent with PGC-1α regulation. Relative mRNA expression of PGC-1α is consistent with ERRα regulation. *mean P<0.05, # mean P>0.05.

The relative mRNA expression of PGC-1α was (1 ± 0.0783), (1.600 ± 0.0435), (0.3227 ± 0.0359) and (0.3018 ± 0.0291) in RL-952, ECC-1, HEC-1A and HEC-1B cells, respectively. The relative mRNA expression of ERRα in the same cells was (1 ± 0.0413), (1.4857 ± 0.0329), (0.2608 ± 0.0356) and (0.163 3± 0.0436), respectively (Figure 2A). Western blot assays showed a similar expression pattern (Figure 2B, Supplementary Figure 1A). Spearman’s correlation analysis showed that the expression of PGC-1α was positively correlated with that of ERRα (r = 0.697, P < 0.01). In line with previous reports, it’s confirmed that PGC-1α and ERRα are overexpressed in RL-952 and ECC-1 cells, and are expressed at low levels in HEC-1A and HEC-1B cells. Thus, we choose the ECC-1 (high expression of PGC-1α and ERRα) and HEC-1A (low expression of PGC-1α and ERRα) cells for further experiments.

In HEC-1A cells, ERRα levels were higher in the OV-ERRα group than in the BLANK and CON groups(BLANK, 1.005 ± 0.0305; CON, 0.985 ± 0.0288; OV-ERRα, 2.884 ± 0.032; P < 0.01). PGC-1α expression increased upon upregulation of ERRα (BLANK, 1.003 ± 0.040; CON, 0.981 ± 0.0324; OV-ERRα, 2.312 ± 0.026; P < 0.01; Figure 2C). Western blot assays confirmed these results (Figure 2D, Supplementary Figure 1B). Next, we treated ECC-1 and HEC-1A cells with XCT790, an ERRα inverse agonist. We observed the downregulation of ERRα levels in XCT790-treated cells compared with the untreated cells or those treated with DMSO (Figure 2E). Interestingly, we observed an ERRα-dependent inhibition of PGC-1α expression following XCT790 treatment (ECC-1: BLANK, 1.001 ± 0.027; DMSO, 0.975 ± 0.043; XCT790, 0.471 ± 0.051; P < 0.01 - HEC-1A: BLANK, 1.003 ± 0.040; DMSO, 0.978 ± 0.0372; XCT790, 0.132 ± 0.067; P < 0.01; Figure 2F). These data show that the expression of PGC-1α is regulated by ERRα. We next investigated whether PGC-1α affects ERRα expression by silencing PGC-1α expression. In ECC-1 cells, PGC-1α expression was significantly lower (89.5 %) in the siPGC-1α group than in the BLANK group, whereas no difference was observed between the BLANK and CON groups (BLANK, 1.001 ± 0.027; CON, 0.998 ± 0.034; siPGC-1α, 0.104 ± 0.0361; P < 0.01). Notably, ERRα levels decreased upon PGC-1α silencing (BLANK, 1.006 ± 0.022; CON, 0.997 ± 0.031; siPGC-1α, 0.0240 ± 0.0111; P < 0.01; Figure 2G). These results were confirmed via western blot analysis (Figure 2H, Supplementary Figure 1C) and suggest that PGC-1α induces ERRα expression in ECC-1 cells.

### Downregulation of PGC-1α and ERRα inhibit the migration and invasion ability of endometrial cancer cells

**Figure 3:**
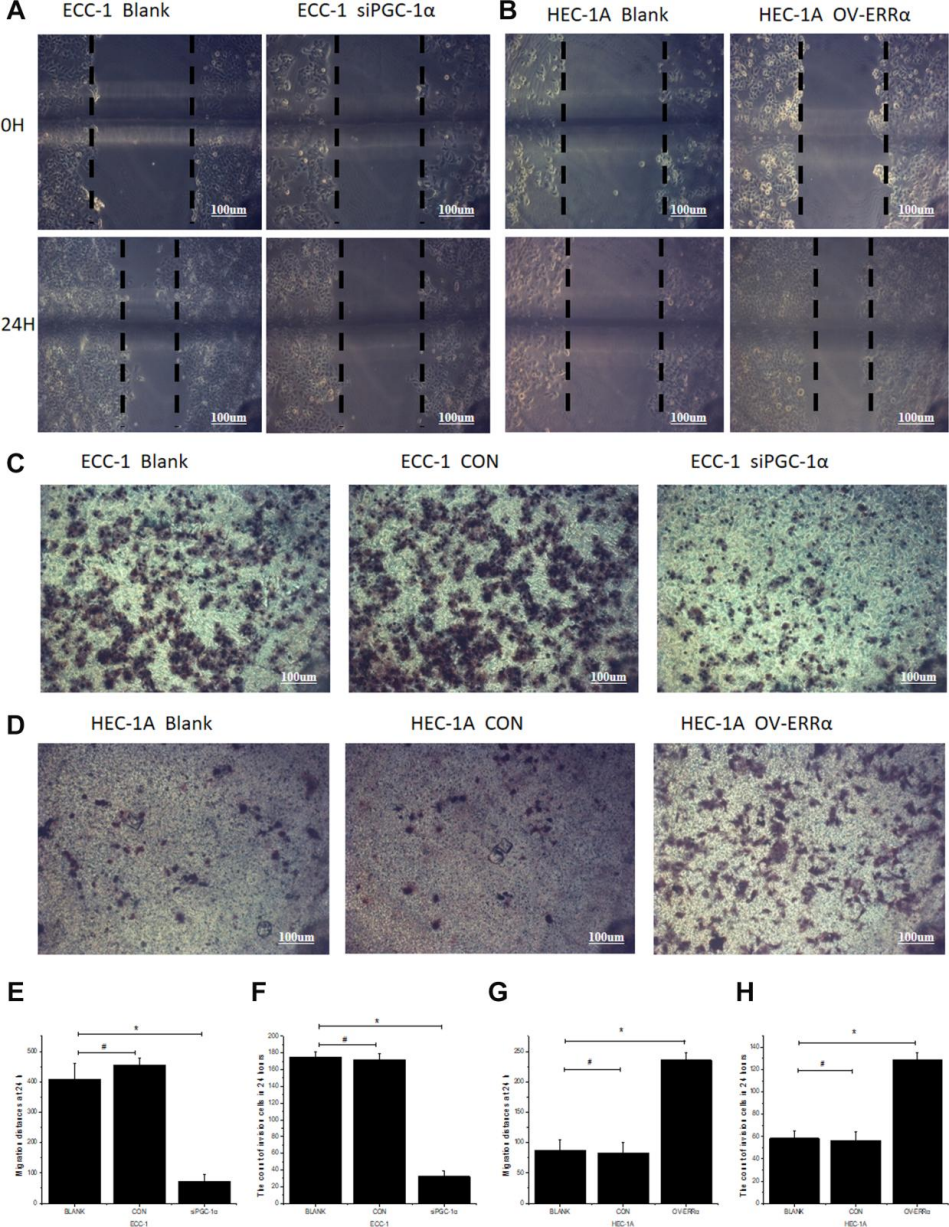
**Regulation of the migration and invasion ability of endometrial cancer cells by PGC-1α and ERRα.** (**A**) Compared with blank groups (Blank), the 24-hour migrated distances of ECC-1 siPGC-1α group were significantly reduced. (**B**) Compared with blank groups (Blank), the 24-hour migrated distances of HEC-1A OV-ERRα group were significantly increased. (**C**) The endometrial cancer cells penetrated the Transwell membrane were significantly reduced in ECC-1 siPGC-1α group compared with Blank group. (**D**) the endometrial cancer cells penetrated the Transwell membrane were significantly increased in HEC-1A OV-ERRα group compared with Blank. Quantification of migration (**E**) and invasion (**F**) of ECC-1 cells. Quantification of migration (**G**) and invasion (**H**) of HEC-1A cells. *mean P<0.05, # mean P>0.05.

We then assessed whether PGC-1α affects cell migration/invasion. In migration assays in ECC-1 cells, the width of the scratch was smaller in the BLANK and CON groups than that in the siPGC-1α group (BLANK, 409.32 ± 50.99; CON, 453.79 ± 22.98; siPGC-1α, 72.898 ± 21.32; F = 200.980, P < 0.001; Figure 3A and 3E). The results in the BLANK and CON groups were comparable (P > 0.05). Person’s correlation analysis showed that PGC-1α and ERRα levels were positively correlated with the migration distance (r_PGC-1α_ = 0.983 and r_ERRα_ = 0.966) (Supplementary Figure 3A, 3B). In invasion assays, the number of invading cells significantly decreased after PGC-1α silencing (BLANK, 174.67 ± 6.532; CON, 172.00 ± 7.176; siPGC-1α, 32.33 ± 6.623; F=847.642, P < 0.001; Figure 3C and 3F). Additionally, the number of invading cells in the BLANK and CON groups were comparable (P > 0.05). Pearson’s correlation analysis showed positive correlation between PGC-1α and ERRα levels and the number of invading cells (r_PGC-1α_ = 0.996 and r_ERRα_ = 0.992) (Supplementary Figure 2C, 2D).

### Enhanced PGC-1α/ERRα expression is associated with distant migration and invasion of endometrial cancer cells

In experiments similar to the ones described above, the migration capability of HEC-1A cells overexpressing ERRα was significantly higher than that of the BLANK and CON groups (BLANK, 87.376 ± 16.905; CON, 82.55 ±1 8.086; OV-ERRα, 235.712 ± 11.832; F = 152.652, P < 0.001; Figure 3B and 3G). The BLANK and CON groups showed similar results (P > 0.05). Person’s correlation analysis showed that PGC-1α and ERRα levels positively correlated with migration distance (r_PGC-1α_= 0.979 and r_ERRα_ = 0.995) (Supplementary Figure 3A, 3B). In invasion assays, the number of invading cells was significantly higher in the OV-ERRα group than in the BLANK or CON groups (BLANK, 58.40 ± 6.269; CON, 56.67 ± 7.23; OV-ERRα, 129.00 ± 6.285; F = 198.471, P < 0.001). The invading capability of cells of the BLANK and CON groups was similar (P > 0.05; Figure 3D and 3H). Pearson’s correlation analysis showed a positive correlation between PGC-1α and ERRα levels and the number of invading cells (r_PGC-1α_ = 0.984 and r_ERRα_ = 0.974)(Supplementary Figure 3C, 3D).

### Inhibition of PGC-1α/ERRα suppresses EMT in endometrial cancer cells

**Figure 4:**
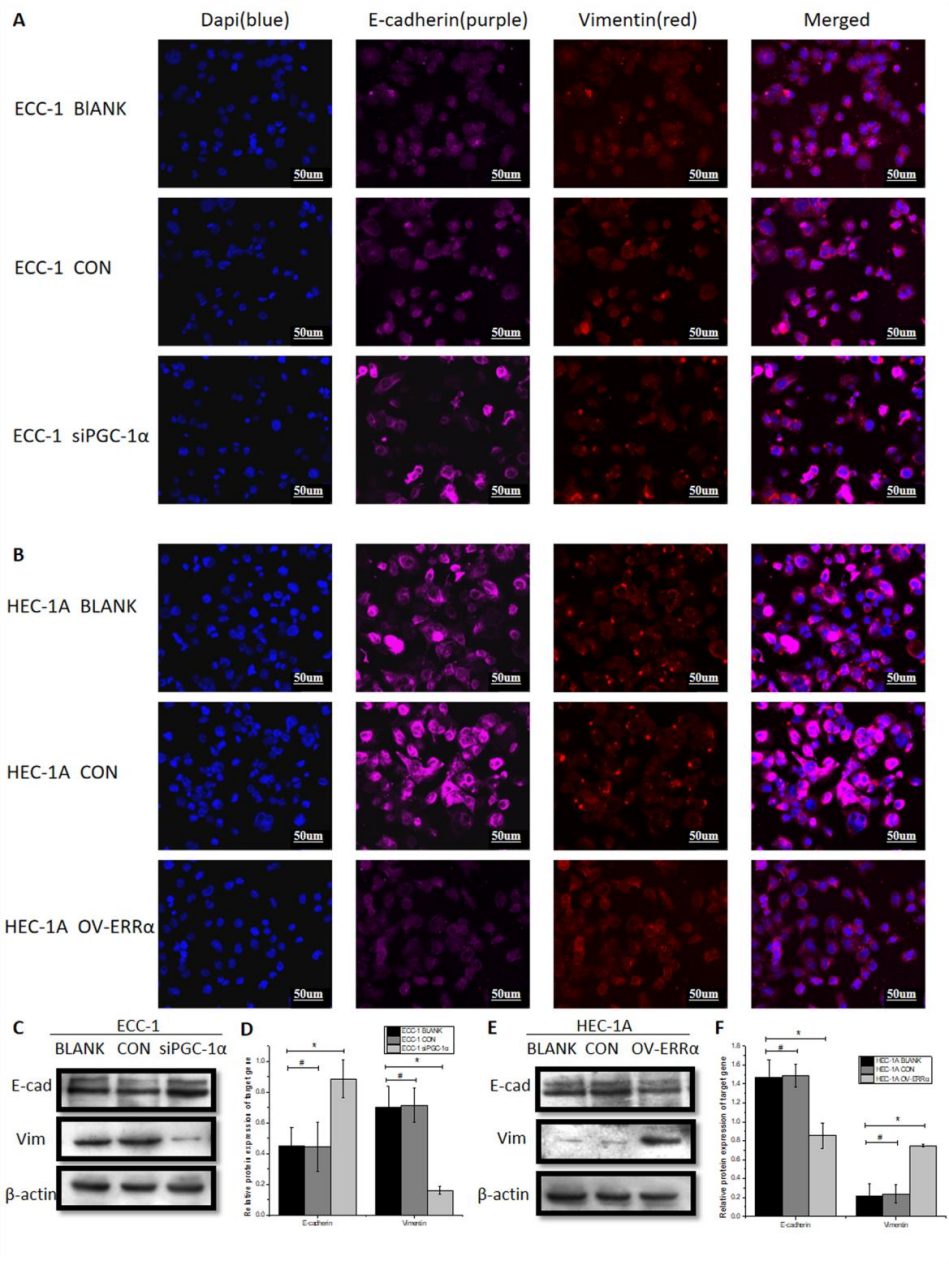
**Up-and down-regulation of PGC-1α and ERRα alter the EMT process in EC cells.** (**A**) the representative immunofluorescence images of E-cadherin (purple) and vimentin (red) in ECC-1cells and siPGC-1α-ECC-1cells. The nucleus is blue. optical microscope and fluorescence microscopic with a magnification of 400. (**B**) the representative immunofluorescence images of E-cadherin (purple) and vimentin (red) in HEC-1A cells and OV-ERRα cells. (**C**) The protein of E-cadherin and vimentin were measured by Western blot analysis in ECC-1cells and siPGC-1α-ECC-1cells. (**D**) Quantification of E-cadherin and vimentin protein in ECC-1 cells. (**E**) The protein of E-cadherin and vimentin were measured by Western blot analysis in HEC-1A cells and OV-ERRα cells. (**F**) Quantification of of E-cadherin and vimentin protein in HEC-1A cells. *mean P<0.05, # mean P>0.05.

Increasing evidence shows that the progression of cancer cell motility is associated with EMT. We therefore hypothesized that PGC-1α plays a positive role in the progression of EMT. Immunofluorescence assays revealed increased expression of the epithelial cell marker E-cadherin (E-cad) and decreased expression of the mesenchymal cell marker vimentin (Vim) in the si-PGC-1α group compared with the BLANK and CON groups (Figure 4A). Western blotting analysis confirmed that inhibition of PGC-1α expression led to increased expression of E-cadherin (BLANK, 0.447 ± 0.125; CON, 0.443 ± 0.162; siPGC-1α, 0.884 ± 0.123; F = 2021.448, P < 0.001) and decreased expression of vimentin (BLANK, 0.700 ± 0.137; CON, 0.712 ± 0.111; siPGC-1α, 0.160 ± 0.026; F = 5533.24, P < 0.001; Figure 4C and 4D).

### Overexpression of PGC-1a/ERRα induces EMT in endometrial cancer cells

In similar experiments, immunofluorescence assays showed that ERRα overexpression in HEC-1A cells was associated with decreased expression of E-cadherin (E-cad) and increased expression of vimentin (Vim) (Figure 4B). Western blotting analysis also revealed that overexpression of ERRα led to decreased expression of E-cadherin (BLANK, 1.447 ± 0.182; CON, 1.486 ± 0.124; OV-ERRα, 0.855 ± 0.135; F = 3087.854, P < 0.001) and increased expression of vimentin (BLANK, 0.215 ± 0.134; CON, 0.236 ± 0.099; OV-ERRα, 0.746 ± 0.014; F = 2992.792, P < 0.001; Figure 4E and 4F). Therefore, the overexpression of ERRα is associated with a more aggressive phenotype.

### Overexpression of ERRα increases TFEB transcription factor activity in endometrial cancer cells

High-throughput protein/DNA array analysis has previously shown that transcription factor EB (TFEB) is downregulated upon ERRα knockdown via PGC-1α. Therefore, we next examined whether TFEB activity was affected by overexpression of ERRα in endometrial cancer cells. TFEB activity was 0.346 ± 0.011 in the BLANK group, 0.345 ± 0.009 in the CON group and 0.469 ± 0.005 in the OV-ERRα group (P < 0.001).

## DISCUSSION

To date, the mechanisms of invasion and metastasis in endometrial cancer have not been fully clarified. Advanced endometrial cancer, which is almost always metastatic, is generally associated with a poor prognosis and eventually causes death. ERRα is involved in the initiation of malignant progression in epithelial cells and is a prognostic marker in various human cancers. Our previous research showed that the expression of ERRα correlates with the high grade and presence of the CA-125 antigen in ovarian tumors, and is thus, associated with reduced survival rates [15]. Matsushima et al. [16] detected ERRα levels in uterine tumors by immunohistochemistry and found that high expression of ERRα is associated with myometrial invasion. Additionally, other studies have found that in various cohorts of patients with breast cancer the mRNA and protein expression of ERRα correlates positively with node status, increased risk of recurrence and metastatic status [17, 18].

ERR proteins play an essential role in regulating the expression of genes involved in cell metabolism, thereby regulating cell proliferation, differentiation, apoptosis and intracellular signaling [13]. However, the transcriptional activity of ERR proteins relies on the presence of coregulatory proteins, especially PGC-1α. Several studies show that high levels of ERRα mRNA and protein in tissues are generally associated with high expression of PGC-1α, and PGC-1α may induce the expression of ERRα mRNA in vivo [19]. Yasuyuki and colleagues have demonstrated in human and mouse cells that ERRα specifically binds to PGC-1α to regulate mitochondrial activity of cells, and the regulation of cellular oxidative phosphorylation induced by PGC-1α is ERRα-dependent [20]. These data suggest that the physical interaction between PGC-1s and the ERR proteins has clear biological significance.

To understand the role of the PGC-1α/ERRα axis in endometrial cancer cells, we silenced PGC-1α in ECC-1 cells using lentivirus-mediated RNA interference. We found that decreased PGC-1α and ERRα expression in cells resulted in significantly reduced cell invasion and migration. Studies have shown that EMT plays an essential role in cancer cell invasion and metastasis [3] and that reduced E-cadherin levels induce EMT and cancer cell migration [21, 22]. Decreased E-cadherin expression indicates the first stage of cancer cell metastasis, and loss of E-cadherin is associated with poor prognosis in patients with cancer [23, 24]. In addition, overexpression of vimentin in MCF7 cells increases cell stiffness, cell motility and directional migration, reorients microtubule polarity, and the EMT phenotype [25].

**Figure 5:**
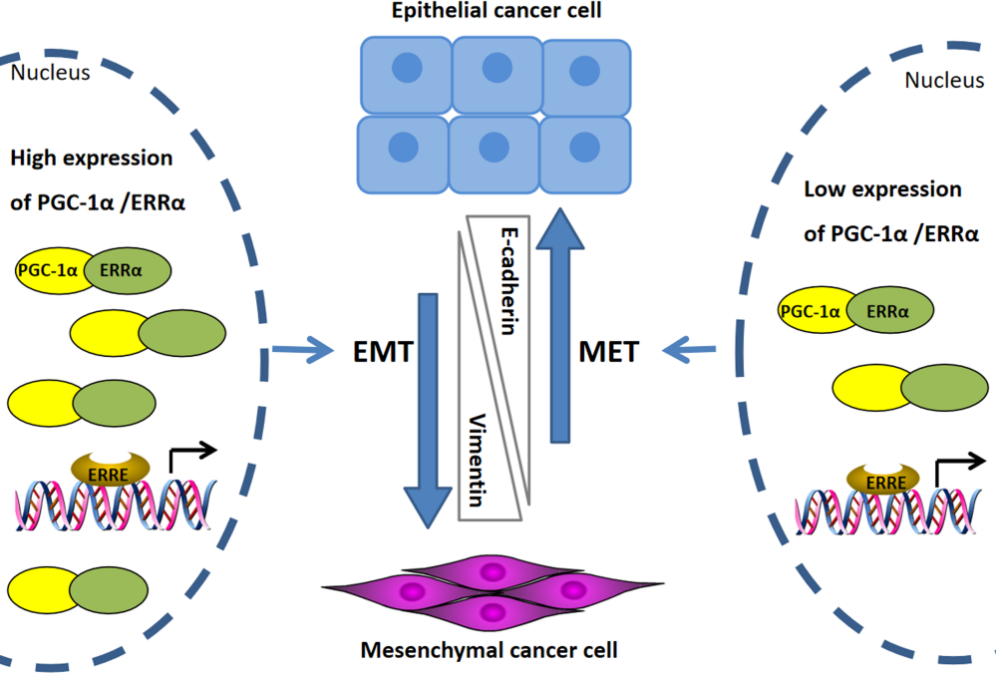
**Mechanism diagram to illustrate the potential role of ERRα/PGC-1α regulating EMT in endometrial cancer.** High expression of PGC-1α/ERRα strengthening EMT phenotypes in endometrial cancer cells, the EMT process accompanied by the decreased expression of the epithelial cell marker E-cadherin, and the increased expression of the mesenchymal cell marker vimentin. Inhibition of PGC-1α/ERRα lead to suppresses the EMT process and upregulation of E-cadherin as well as downregulation of vimentin.

This study showed that silencing of PGC-1α, impairing ERRα activation, suppressed the migration and invasion of endometrial cancer cells. This process relied on the downregulation of E-cadherin and upregulation of vimentin. On the other hand, overexpression of ERRα, induced PGC-1α expression and enhanced cancer cell migration and invasion by strengthening EMT phenotypes (Figure 5). Therefore, we speculated that PGC-1α and ERRα are interdependent in the induction of EMT. Research by Taiwan scholars have shown that PGC-1α and ERRα synergistically promote the expression of multiple nuclear-encoding genes associated with mitochondrial fusion. Increased PGC-1α and ERRα expression, induced by high glucose, mediated mitochondrial pathways and significantly induced EMT in OVCAR-3 cells. Furthermore, reduced levels of ERRα and PGC-1α levels upon AEPP treatment lead to downregulations of cell survival and EMT in the same cells [26]. Lam et al. studied the correlation between ERRα and changes in biological functions in ovarian cancer cells and ERRα role in EMT. The authors found that ERRα overexpression correlated with poor outcome in ovarian cancer. Targeted inhibition of ERRα suppressed EMT through inhibition of E-cadherin expression. Additionally, ERRα increased snail expression by increasing gene transcription and mRNA stability, thereby promoting EMT in cancer cells [27]. These results confirm that PGC-1α and ERRα are critical positive regulators of EMT and inducers of cancer metastasis.

TFEB induces the expression of genes involved in autophagy and lysosomal biosynthesis, positively enhances lysosomal fat degradation, lipolysis, and intracellular fatty acid oxidation [28]. Recently, Blessing et al. found that TFEB promotes prostate cancer progression through regulation of SQSTM1 equivalent [29]. Additionally, Jing et al. found that the high expression of TFEB is positively correlated with the aggressiveness of colon cancer. TFEB regulates autophagy in colon cancer cells by promoting Beclin1 expression, resulting in tumor cell metastasis [30]. Notably, in 2012, Tsunemi et al. showed that the ability of PGC-1α to clear mutant Huntingtin was dependent on TFEB, suggesting that PGC-1α and TFEB are potential therapeutic targets for Huntington and other neurodegenerative diseases [31]. Furthermore, Grassi et al. found that autophagy affects the differentiation of liver cells, which causes imbalance of EMT/MET in liver cells, leading to changes in cell invasion ability [32]. Our result showed that overexpression of ERRα increases TFEB activity. Therefore, we speculated that the PGC-1α/ERRα axis participates in EMT by regulating TFEB.

In summary, our study identifies a novel role for PGC-1α and ERRα as positive regulators of EMT. Our data suggest that disruption of the PGC-1α/ERRα signaling could serve as a new strategy for reversing EMT and inhibit endometrial cancer invasion and migration.

## MATERIALS AND METHODS

### Ethics committee approval

The study was conducted in accordance with ethical standards, the Declaration of Helsinki, and national and international guidelines, and has been approved by the Ethics Committee of Fujian Maternity and Child Health Hospital affiliated with Fujian Medical University (No.2018-014). An informed consent was obtained from all patients.

### Study population and tissues

A total of 81 endometrial carcinoma and 40 normal endometrium samples with related clinical data were obtained from the patients who underwent surgical therapy in Fujian provincial Maternity & Children’s Health Hospital of Fujian Medical University, China. None of the patients received any chemotherapy, radiation, or hormonal therapy before surgery. An informed consent was obtained from all patients. This research protocol was approved by the Ethics Committee of Fujian Maternity and Child Health Hospital affiliated with the Fujian Medical University (No.2018-014).

### Immunohistochemistry

All tissues were assembled into a tissue chip. Immunostaining for ERRα and PGC-1α was performed according to standard procedures. Rabbit polyclonal anti-ERRα (dilution 1:100; Abcam, UK) and rabbit polyclonal anti-PGC-1 alpha-N-terminal (dilution 1:200; Abcam) antibodies were used. The percentage of positive cells was scored as 0 (cells < 5%), 1 (5% to 25%), 2 (26% to 50%), 3 (51% to 75%), and 4 (76% to 100%). Positive staining intensity was scored as 0 (no staining), 1 (weak staining), 2 (moderate staining), and 3 (strong staining). The expression level of ERRα and PGC-1α was measured by the immunoreactive score (IRS) using the algorithm: IRS = Si × Pi (where Si and Pi represent the intensity and percentage of cells with each intensity, respectively). Samples were divided into four groups based on their IRS: 0, negative (-); 1-4, weakly positive (+); 5-8, positive (++); 9-12, strongly positive (+++).

### Cell culture and treatment with XCT790

The endometrial cancer cell lines RL-952, ECC-1, HEC-1A and HEC-1B were purchased from the American Typical Culture Collection (ATCC, Rockville, USA). The cells were grown as previously described [33]. Cells treated with XCT790 (Sigma-Aldrich, St. Louis, MO, USA) were incubated in phenol red-free medium (Thermo Fisher) containing 1% Serum Replacement 2 (Sigma-Aldrich). ECC-1 and HEC-1A EC cells were incubated with 10 μM XCT790 (in dimethyl sulfoxide [DMSO]; Sigma-Aldrich) or DMSO (control) for 24 h.

### Quantitative PCR (qPCR)

Total RNA was isolated according to the manufacturer’s protocol (Invitrogen, USA, Thermal), as previously described [33]. The mRNA was transcribed into cDNA using an Access RT-PCR System (Promega, USA). qPCR assays were performed using a LightCycler^®^ 480 SYBR Green I Master Mix (Roche, Germany). The following primers, synthesized by Sanggong Biotech (Shanghai, China), were used: PGC-1α, forward 5′-GAC ACA ACA CGG ACA GAA-3′ and reverse 5′- CAC AGG TAT AAC GGT AGG TAA -3′ (PCR product, 121 bp); ERRα, forward 5′- ACC GAG AGA TTG TGG TCA CCA -3′ and reverse 5′- CAT CCA CAC GCT CTG CAG TAC T- 3′ (101 bp); and GAPDH, forward 5′- GCA CCG TCA AGG CTG AGA AC- 3′ and reverse 5′- TGG TGA AGA CGC CAG TGG A -3′ (138 bp). The target gene mRNA levels were quantified using the comparative method (2^−ΔΔCT^ method) and normalized to GAPDH expression.

### Western blotting

Whole-cell proteins were extracted according to the manufacturer’s protocol (Clontech, Palo Alto, USA) and their concentration was determined using an ELISA kit (Pierce), as previously described [33]. Thirty micrograms of whole-cell protein lysate was loaded to each lane of an 8%-polyacrylamide gel. Proteins were blotted onto nitrocellulose membranes. Membranes were incubated with a rabbit monoclonal antibody specific to ERRα (1:500; CST), PGC-1α (1:1,000; CST), or vimentin (1:1,000; CST) or E-cadherin (1:1,000; CST) in blocking buffer, followed by incubation with an alkaline phosphatase-conjugated secondary antibody (1:1,000; Abcam). Immunoreactive bands were visualized using the CDP star RTU luminescence system (Tropix).

### Lentivirus-mediated silencing of PGC-1α and overexpression of ERRα

A lentiviral vector expressing small interfering RNAs (siRNA) targeting PGC-1α and named siRNA-PGC-1α (siPGC-1α) was constructed. The following siRNA target sequence in the PGC-1α gene (GenBank accession No. NM_013261) was selected: siPGC-1α, 5′- CAA CTT TAT CTC TTC CTC TGA -3′. A universal sequence (PSC-NC: 5′- TTC TCC GAA CGT GTC ACG T -3′ named CON) was used as the negative control. The lentiviral vector used to overexpress ERRα (named ESRRA, GenBank accession NM_004451) or the universal sequence (negative control) were purchased from Genechem (Shanghai, China). Three groups of cells were analyzed: cells transduced with lentiviruses expressing siRNAs targeting PGC-1α (siPGC-1α group) or overexpressing ERRα (OV-ERRα group), cells transduced with empty lentiviruses (CON group), and untreated cells (BLANK group). Lentivirus-based vectors carry the green fluorescent protein (GFP) gene (GV115, Genechem, Shanghai, China). After transduction for 72 h, GFP expression was detected to calculate the transduction efficiency (Supplementary Figure 1D–1E). EC cells were transduced at a multiplicity of infection (MOI) of 100. After 72 h of transduction, GFP expression was detected to calculate the infection efficiency.

### In vitro cellular scratch assays

Cells were grown to confluence in 6-well plates and a 200-μL tip was used to introduce a scratch in the monolayer. The scratch areas in the wells were washed with PBS and 1 mmol/L R-flurbiprofen until the cells in those areas were removed thoroughly and imaged at 0 and 24 h post-scratching. The horizontal migration rate was calculated using the following formula: (width_0 h_ − width_24 h_)/width _0 h_ × 100% [34].

### Transwell chamber invasion assays

Matrigel™ Basement Membrane Matrix (50 μL; BD, USA) was added to a Millicell Hanging Cell Culture Insert (Millipore, USA) to coat the membrane. Two hundreds microliters of cell suspension containing 0.5% FBS (5.0 × 10^5^ cells/mL) were added to the insert, placed in 24-well plates containing 1,300 μL of DMEM supplemented with 10% FBS. After incubation for 24 h, non-invading cells on the top of the filter were removed with a cotton swab, and the filters were fixed with methanol and stained with crystalline violet. The filters were removed from the inserts and mounted onto slides for imaging and quantification as described in a previous study [34].

### TFEB transcription factor assay

Nuclear and cytoplasmic cell extracts were prepared using a Nuclear Extract kit. One hundred microliters of nuclear extract was added into a 96-well plate with immobilized oligonucleotides containing TFEB consensus binding sites. A positive control for TFEB activation was set, and 20 μL complete lysis buffer served as the blank. A diluted primary antibody (100 μL) was incubated for 1 h at room temperature. After three washes, an HRP-labeled secondary antibody was incubated. After 50 μL stop solution was added, the absorbance was measured by a microplate reader (RayBio).

### Cell immunofluorescence

Cells were fixed with 4% paraformaldehyde, permeabilized with 0.5% Triton X-100, and incubated with blocking buffer samples, and then with a rabbit monoclonal antibody specific to vimentin (1:100 dilution, CST) and mouse monoclonal antibody specific to E-cadherin (1:50 dilution, CST) at 4 °C overnight. After extensive wash, secondary anti-rabbit IgG conjugated with Alexa 647(CST) or anti-mouse IgG conjugated with Alexa 594 (Proteintech) were incubated. The samples were then fixed and analyzed via confocal microscopy. Cell fluorescence intensity was quantified with Image J.

### Statistical analysis

Statistical analysis was performed using the average results of three experiments under identical conditions. Numerical data are presented as the mean ± SD. Differences between two means were compared using Student’s t-test, and related parameters were analyzed using Pearson’s correlation. Correlation coefficients for graded data were obtained using spearman correlation analysis. Data were analyzed using the SPSS 17.0 software for Windows (SPSS Inc., Chicago, IL, USA). Differences were considered significant at P< 0.05.

## Supplementary Material

Supplementary Figures
