# Supplementary material for: PGC-1α and ERRα in patients with endometrial cancer: a translational study for predicting myometrial invasion
Source: Aging (Albany NY). 2020 Sep 13;12(17):16963–80. doi: 10.18632/aging.103611 (PMC7521515; doi:10.18632/aging.103611)
Supplement: Supplementary Figures [file aging-12-103611-s001..pdf]

SUPPLEMENTARY FIGURES

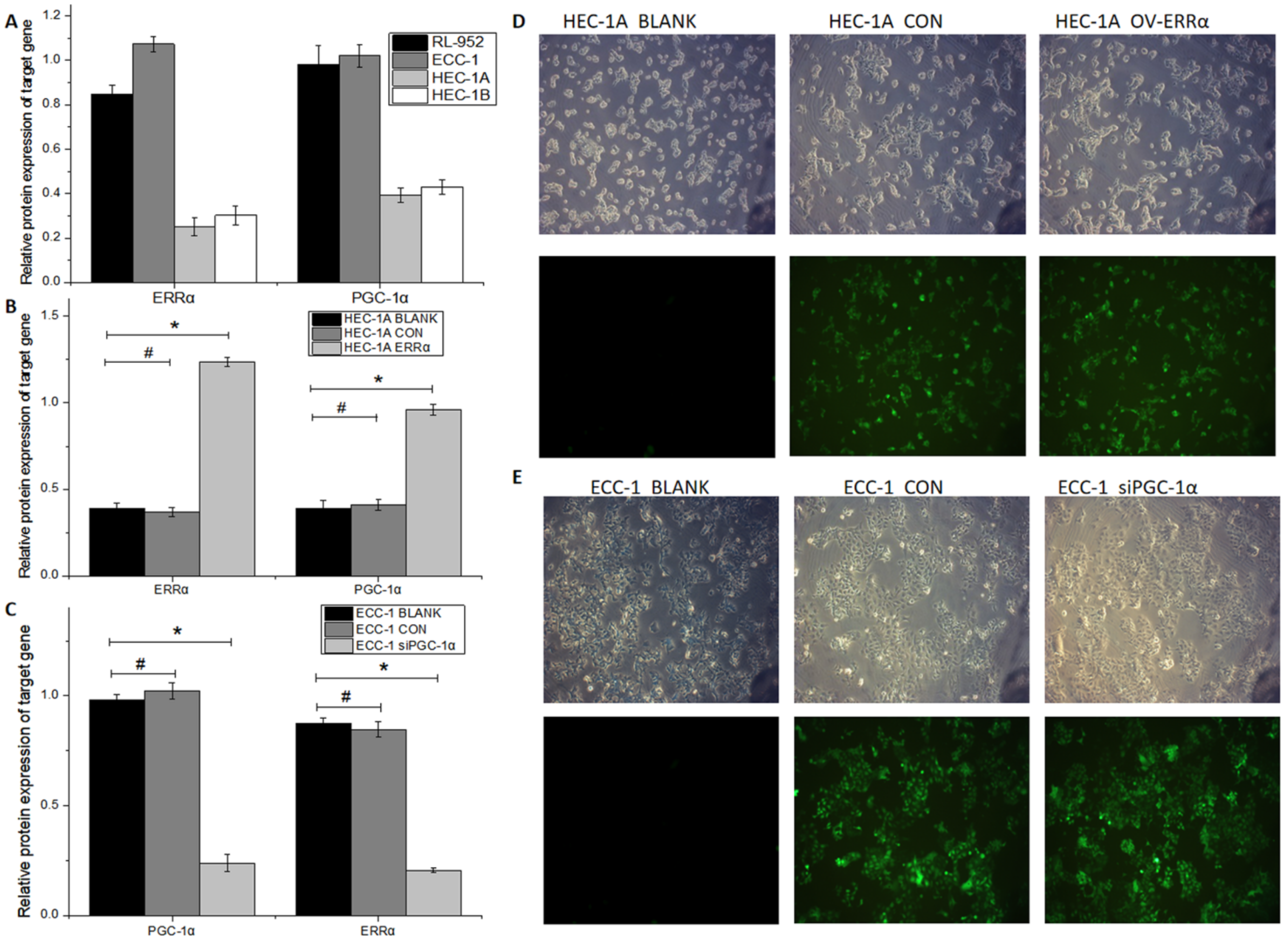

Supplementary Figure 1. (**A**) Quantification data of PGC-1α and ERRα protein expression in RL-952, ECC-1, HEC-1A and HEC-1B endometrial cancer cells. (**B**) Quantification data of PGC-1α and ERRα protein levels after the infection with lentivirus targeted on OV-ERRα in HEC-1A. (**C**) Quantification data of PGC-1α and ERRα protein expression after the infection with PGC-1α-siRNA in ECC-1 cell. Infection with lentivirus vector targeted on overexpression of ERRα in HEC-1A (**D**) and silencing of PGC-1α in ECC-1 (**E**) were observed by optical microscope and fluorescence microscopic with a magnification of 400. *mean P<0.05, # mean P>0.05

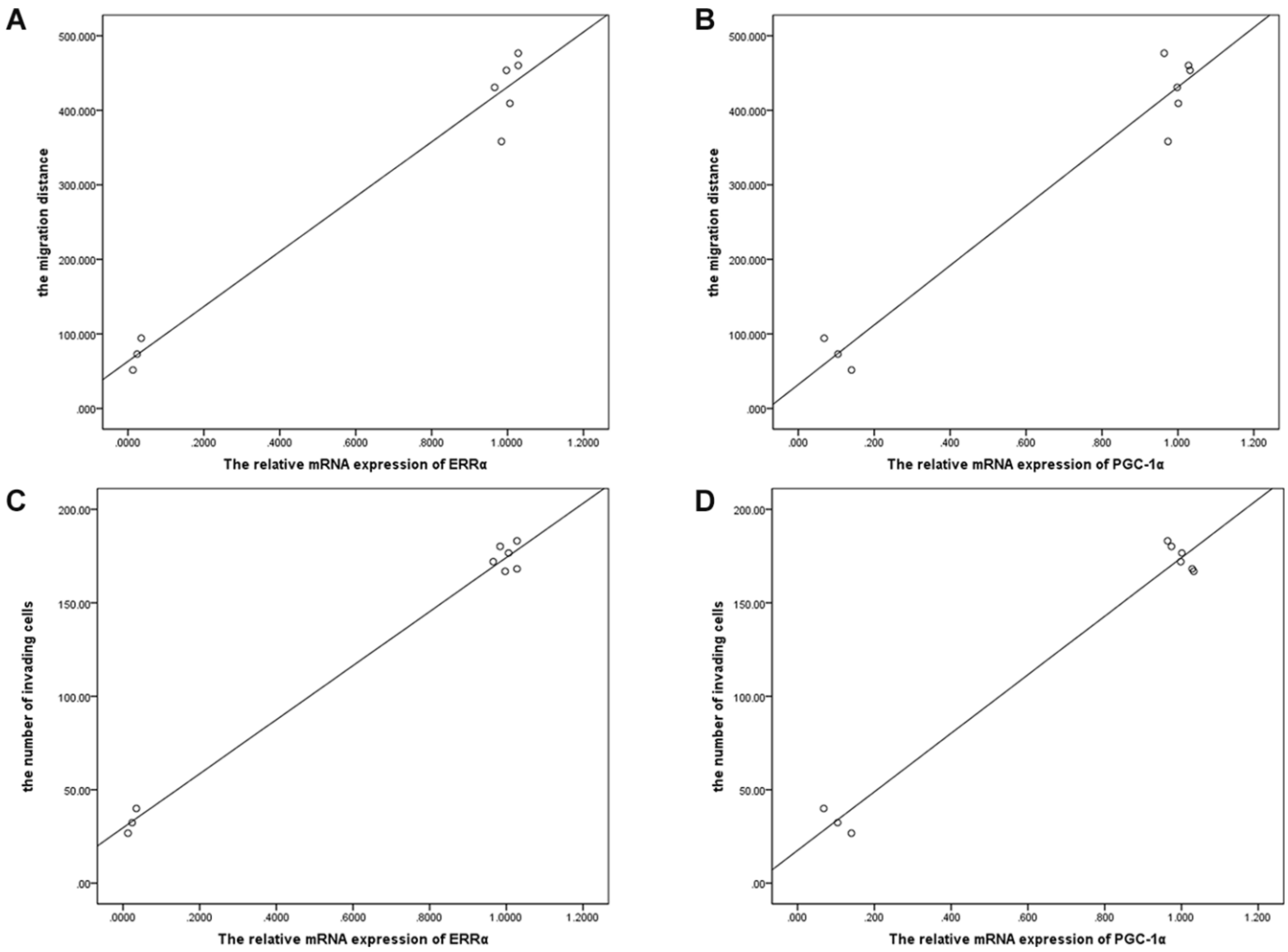

Supplementary Figure 2. Correlation analysis for ERRα (**A**), PGC-1α (**B**) levels and the migration distance after PGC-1α knockdown in ECC-1. Correlation analysis for ERRα (**C**), PGC-1α (**D**) levels and the number of invading cells after PGC-1α knockdown in ECC-1.

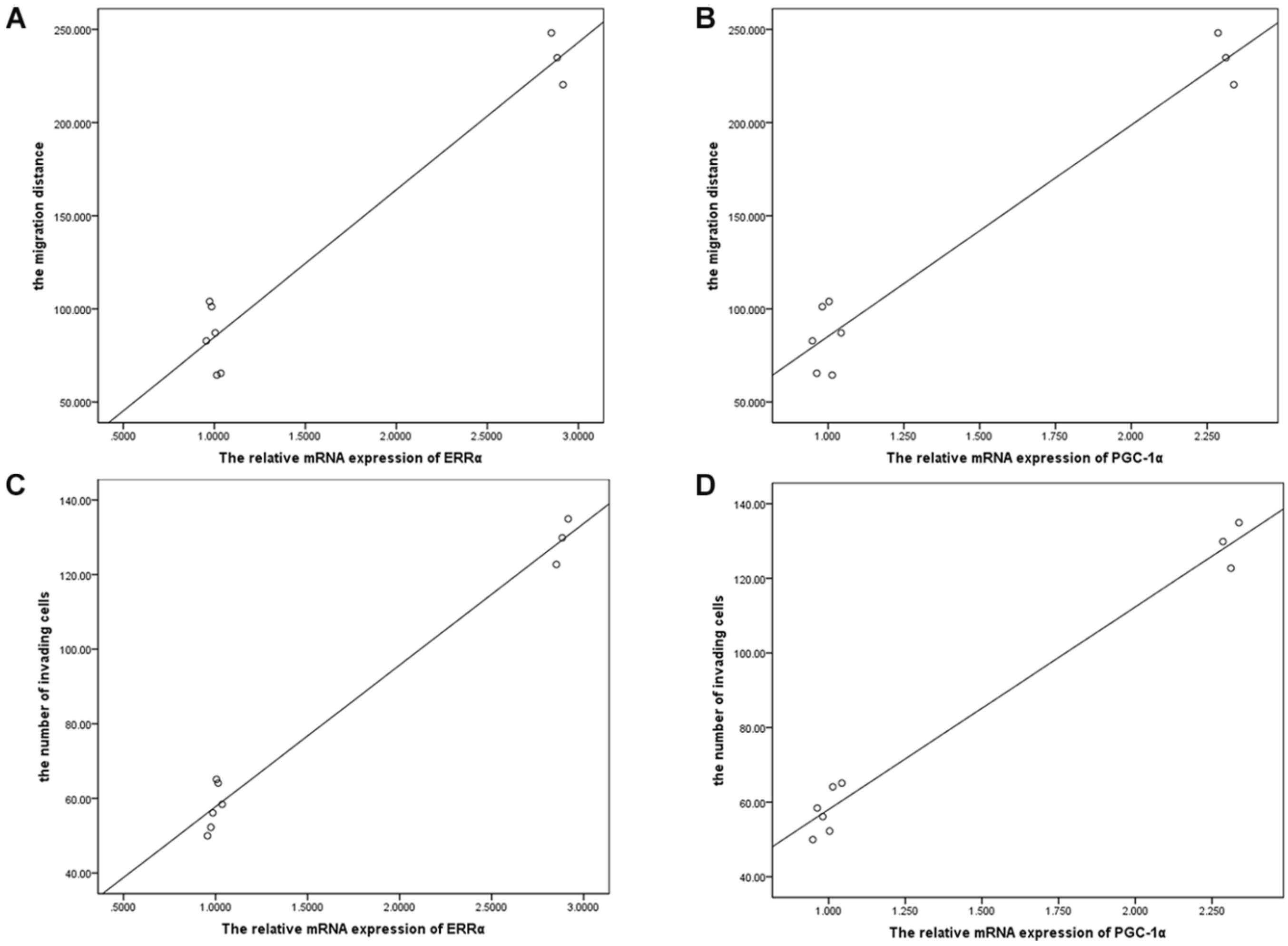

Supplementary Figure 3. Correlation analysis for ERRα (**A**), PGC-1α (**B**) levels and the migration distance after ERRα overexpression in HEC-1A. Correlation analysis for ERRα (**C**), PGC-1α (**D**) levels and the number of invading cells after ERRα overexpression in HEC-1A.
